# Towards targeted drugs and next generation of nanomedicines

**DOI:** 10.3762/bjnano.17.41

**Published:** 2026-05-06

**Authors:** Anna Salvati, Silvia Giordani, Wolfgang J Parak

**Affiliations:** 1 University of Groningen, Groningen Research Institute of Pharmacy, Groningen, The Netherlands; 2 School of Chemical Sciences, Dublin City University, Glasnevin, Dublin, Ireland; 3 Center for Hybrid Nanostructures (CHyN), University of Hamburg, Hamburg, Germany

**Keywords:** cellular uptake, drug delivery, intracellular trafficking, nanomedicine, nanoparticles, tumor microenvironment

Nanomedicine is dedicated to the application of nanotechnology in the medical field. Nanosized materials are intended for delivering drugs to their target, hence improving drug efficacy while minimizing unwanted side effects. At the same time, some nanomaterials can provide new therapeutic modalities themselves and could also be used for diagnosis and imaging. Multiple drugs and therapies can be combined within the same carrier particle to potentially create synergistic effects for the treatment of complex pathologies [1–3].

Since the approval of the first liposomal drug for cancer therapy, Doxil [4], multiple nanomedicines have reached the market, including lipid nanoparticles for RNA delivery used worldwide for the vaccines against SARS-CoV-2 infection during the COVID-19 pandemic [5–7]. While the successes of these technologies are clear, extensive research is still focused on further improving nanomedicine efficacy toward the development of the next generation of nanomedicines.

From a semantic point of view, one might argue that the first drug carriers that reached the clinics, such as liposomes and protein-based drug carriers (e.g., Doxil and Abraxane, respectively) have been investigated for drug delivery long before the term "nanomedicine" was coined, at the end of the last century, and became popular. The same is true for colloids, which were investigated also long before they were called "nanoparticles". On the other hand, such argument leaves out the fact that lipid-based drug carriers and colloids used today in nanomedicine are quite distinct from the ones from the past. For instance, the lipid nanoparticles used for vaccination are composed of a complex mixture of different lipids, all of which have a particular function. Additionally, inorganic nanoparticles can be made with controlled size and shape out of many different materials. Nanotechnology has also made standardized characterization and quality controls available, which were decisive for the development towards clinical use. Last but not least, the field of nanosafety/nanotoxicology has set focus on the importance of counterbalancing the benefits and risks of the use of nanomaterials for potential clinical applications. Hence, while the first clinically approved nanomedicines such as Doxil or Abraxane used "natural nanoparticles" (i.e. lipid vesicles and proteins) for changing the biodistribution of pharmaceutical drugs, there is hope that fully synthetic engineered nanoparticles may be used in the future. There is, thus, a large potential for future development.

Bioavailability is an important factor for pharmaceutical agents. Here nanoparticles have the great advantage that their physicochemical properties can be largely tailored. A hydrophobic drug can be made more water soluble by linking it to a hydrophilic nanoparticle. However, as with conventional drugs, one factor which is still limiting nanomedicine efficacy is their targeting capacity, especially in applications requiring extra-hepatic delivery. In fact, upon administration, most nanomaterials are sequestered by the liver, and only a small fraction of the injected nanoparticles reaches other organs [1,8]. For instance, in the context of tumor targeting, only less than 1% of injected nanoparticles reach the tumor [8]. While this and other similar works in the literature have sparked a debate on the success of the field [9–12], the COVID-19 pandemic and approval of the first RNA vaccines have clearly showcased the potential of nanomedicine [7,13–14]. Still, it is clear that more research is needed to further improve nanomedicine efficacy.

In the context of targeting, multiple strategies have been developed and are used to promote the delivery of nanomedicines to their targeted site. The so-called enhanced permeation and retention (EPR) effect has long been exploited for passive targeting of drug carriers to tumors via leaky tumor blood vessels (Doxil is indeed an example of passively targeted nanomedicine, among several others [15–17]). The success of this strategy has also been debated, since not every tumor can be reached by passive targeting, and the presence of leaky blood vessels can strongly vary within individual patients and in different areas within the same tumor. This has suggested that patient stratification may be required to select those who may truly benefit of passive targeting [15,18–19]. Also, results from animal models often cannot be translated to humans, sometimes simply due to too high tumor weight/body weight ratios: while the EPR effect may help to target a tumor of 1 cm in a "small" mouse, it may fail to target tumors below 1 mm of size in a "big" human. At the same time, recent findings suggested that nanoparticles may be able to reach tumor tissue also via active mechanisms of uptake and transport across tumor endothelial cells, potentially opening up new ways to reach tumors with drugs [20–22].

Next to passive targeting, active targeting strategies are also being investigated, where drugs and drug carriers are modified via the addition of ligands specifically recognizing receptors overexpressed at the targeted cells. While few antibody–drug conjugates have been approved for clinical use [23], active targeting still remains highly challenging, and active targeted drug carriers have not yet achieved clinical approval [15,24–25]. While research is still ongoing to understand factors limiting active targeting and how to design active targeted drug carriers, next to passive and active targeting, other novel targeting strategies are emerging and are being investigated to be able to overcome their current limits.

One strategy to achieve targeting which is attracting extensive attention is that of using materials responsive to endogenous or exogenous stimuli [26]. By designing materials that respond to stimuli, treatments can be targeted to the area of interest, even if the material itself may also reach other areas. Endogenous stimuli are characteristics of the targeted site that distinguish it from the healthy tissue, such as the lower pH or lower oxygen content of certain tumor areas [15]. External stimuli include light or other forms of radiation, magnetic fields, and ultrasounds. These can be applied externally only at the targeted area, hence providing both spatial and temporal control of the therapy [26].

In parallel to this, more recently, endogenous targeting is emerging as another potential strategy to affect nanoparticle distribution and reach the target organ [24,27]. Endogenous targeting refers to the capacity of targeting acquired by nanomedicines after administration, upon adsorption on their surface of specific endogenous biomolecules. In fact, while nanomedicines are usually modified to prevent protein binding in order to avoid immune cell recognition and clearance, hence prolonging nanomedicine circulation time, in some cases the adsorbed endogenous proteins may provide this endogenous targeting capacity [28–29]. First evidence of this type of targeting has been reported for Onpattro, the first lipid nanoparticle approved in the clinics for RNA delivery (in this case short interfering RNA). It was reported that this nanomedicine reaches the hepatocytes because of the adsorption of apolipoprotein E on its surface once administered, driving accumulation in the liver and promoting interaction with the low density lipopoprotein receptor (LDLR) on the hepatocytes [29]. Other examples of nanomedicines exploiting endogenous targeting are the so-called selective organ-targeting (SORT) lipid nanoparticles, which adsorb different corona proteins on their surface depending on their charge, enabling targeting to either the liver, the lung, or the spleen [30]. While stimuli-responsive nanomedicines and endogenous targeting are investigated as potential new strategies to achieve targeting, other research topics focus on alternative administration routes for specific applications, such as pulmonary delivery for lung diseases and nose-to-brain delivery as a means of reaching the brain [31–32]. Overall, while we are also gaining a better understanding of how nanomaterials are processed at organism and cell levels, research is exploring several new directions for advancing the development of novel nanomedicines with improved efficacy.

Within this context, this thematic issue collects different contributions on some of the latest developments in nanomedicine and emerging targeting strategies for the development of next generation nanomedicines. The thematic issue also aimed to include contributions emerging from the Beilstein Nanomedicine Symposium [33] which took place in September 3–5, 2024 in Rüdesheim, Germany and focused on similar topics, such as: advances of nanomedicines and their potential in precise targeting therapies, personalized medicine and reliable pre-clinical assessments, synthesis and functionalization of nanobiomaterials, nanotechnology applied to therapy and medical diagnostics, and pharmaceutical nanotechnology.

The collected articles and contributions provide a broad overview on recent advances in the field within the aforementioned topics. Additionally, it includes several reviews on the current development of specific classes of nanomaterials, nanomedicines applied to specific diseases, and fundamental understanding of how nanomedicines are processed by cells and in the body.

As a conclusion one can say that nanomedicine is in its infancy steps, where first formulations are in clinical use. There are still big hurdles, such as targeting and crossing of biological barriers (e.g., endosomal escape) which need to be better tackled in the future. For this, apart from applied research, a lot of fundamental basic research problems need to be addressed [34–35]. Should this be possible, the nanomedicines would reach the next step of maturity.

Anna Salvati, Silvia Giordani, and Wolfgang J. Parak

Groningen, Dublin, and Hamburg, March 2026

## Data Availability

Data sharing is not applicable as no new data was generated or analyzed in this study.

## References

[1] Blanco E, Shen H, Ferrari M (2015). Nat Biotechnol.

[2] Shi J, Kantoff P W, Wooster R, Farokhzad O C (2017). Nat Rev Cancer.

[3] van der Meel R, Sulheim E, Shi Y, Kiessling F, Mulder W J M, Lammers T (2019). Nat Nanotechnol.

[4] Barenholz Y (2012). J Controlled Release.

[5] Anselmo A C, Mitragotri S (2019). Bioeng Transl Med.

[6] Anselmo A C, Mitragotri S (2021). Bioeng Transl Med.

[7] Weiss C, Carriere M, Fusco L, Capua I, Regla-Nava J A, Pasquali M, Scott J A, Vitale F, Unal M A, Mattevi C (2020). ACS Nano.

[8] Wilhelm S, Tavares A J, Dai Q, Ohta S, Audet J, Dvorak H F, Chan W C W (2016). Nat Rev Mater.

[9] Lammers T, Kiessling F, Ashford M, Hennink W, Crommelin D, Storm G (2016). Nat Rev Mater.

[10] Venditto V J, Szoka F C (2013). Adv Drug Delivery Rev.

[11] van der Meel R, Lammers T, Hennink W E (2017). Expert Opin Drug Delivery.

[12] McNeil S E (2016). Nat Rev Mater.

[13] Friedrichs S, Bowman D M (2021). Nat Nanotechnol.

[14] Chung Y H, Beiss V, Fiering S N, Steinmetz N F (2020). ACS Nano.

[15] Danhier F, Feron O, Préat V (2010). J Controlled Release.

[16] Fang J, Nakamura H, Maeda H (2011). Adv Drug Delivery Rev.

[17] Matsumura Y, Maeda H A (1986). Cancer Res.

[18] Chauhan V P, Jain R K (2013). Nat Mater.

[19] Miller M A, Gadde S, Pfirschke C, Engblom C, Sprachman M M, Kohler R H, Yang K S, Laughney A M, Wojtkiewicz G, Kamaly N (2015). Sci Transl Med.

[20] Sindhwani S, Syed A M, Ngai J, Kingston B R, Maiorino L, Rothschild J, MacMillan P, Zhang Y, Rajesh N U, Hoang T (2020). Nat Mater.

[21] Wu J L Y, Ji Q, Blackadar C, Nguyen L N M, Lin Z P, Sepahi Z, Stordy B P, Granda Farias A, Sindhwani S, Ngo W (2025). Nat Nanotechnol.

[22] Li X, Hu Y, Zhang X, Shi X, Parak W J, Pich A (2024). Nat Commun.

[23] Fu Z, Li S, Han S, Shi C, Zhang Y (2022). Signal Transduction Targeted Ther.

[24] Dilliard S A, Siegwart D J (2023). Nat Rev Mater.

[25] Muro S (2012). J Controlled Release.

[26] Mura S, Nicolas J, Couvreur P (2013). Nat Mater.

[27] Akinc A, Querbes W, De S, Qin J, Frank-Kamenetsky M, Jayaprakash K N, Jayaraman M, Rajeev K G, Cantley W L, Dorkin J R (2010). Mol Ther.

[28] Salvati A (2024). Curr Opin Biotechnol.

[29] Akinc A, Maier M A, Manoharan M, Fitzgerald K, Jayaraman M, Barros S, Ansell S, Du X, Hope M J, Madden T D (2019). Nat Nanotechnol.

[30] Cheng Q, Wei T, Farbiak L, Johnson L T, Dilliard S A, Siegwart D J (2020). Nat Nanotechnol.

[31] Feng X, Shi Y, Zhang Y, Lei F, Ren R, Tang X (2024). Int J Nanomed.

[32] Wang Z, Xiong G, Tsang W C, Schätzlein A G, Uchegbu I F (2019). J Pharmacol Exp Ther.

[33] (2026). Beilstein Nanotechnology Symposium: The next generation of nanomedicines: Formulation to precision therapies.

[34] Feliu N, Parak W J (2024). Science.

[35] Francia V, Montizaan D, Salvati A (2020). Beilstein J Nanotechnol.

